# Anatomy and Histochemistry of the Roots and Shoots in the Aquatic Selenium Hyperaccumulator *Cardamine Hupingshanensis* (Brassicaceae)

**DOI:** 10.1515/biol-2019-0035

**Published:** 2019-07-23

**Authors:** Jiqian Xiang, Jiajia Ming, Hongqing Yin, Yunfen Zhu, Yajie Li, Lan Long, Ziyun Ye, Haiying Wang, Xiaoe Wang, Fan Zhang, Yongkang Yang, Chaodong Yang

**Affiliations:** 1 Engineering Research Center of Ecology and Agriculture Use of Wetland, Ministry of Education, Yangtze University, Jingzhou,434025 China; 2 Hubei Selenium Industry Technology Research Institute, Enshi 454000 China

**Keywords:** air spaces, *Cardamine hupingshanensis*, collenchyma, endodermis, lignified Φ thickenings, sclerenchyma ring

## Abstract

The perennial selenium (Se) hyperaccumulator *Cardamine hupingshanensis* (Brassicaceae) thrives in aquatic and subaquatic Se-rich environments along the Wuling Mountains, China. Using bright-field and epifluorescence microscopy, the present study determined the anatomical structures and histochemical features that allow this species to survive in Se-rich aquatic environments. The roots of *C. hupingshanensis* have an endodermis with Casparian walls, suberin lamellae, and lignified secondary cell walls; the cortex and hypodermal walls have phi (Φ) thickenings; and the mature taproots have a secondary structure with a periderm. The stems possess a lignified sclerenchymal ring and an endodermis, and the pith and cortex walls have polysaccharide-rich collenchyma. Air spaces are present in the intercellular spaces and aerenchyma in the cortex and pith of the roots and shoots. The dense fine roots with lignified Φ thickenings and polysaccharide-rich collenchyma in the shoots may allow *C. hupingshanensis* to hyperaccumulate Se. Overall, our study elucidated the anatomical features that permit *C. hupingshanensis* to thrive in Se-rich aquatic environments.

## 1 Introduction

*Cardamine hupingshanensis* (Brassicaceae) is a perennial aquatic and subaquatic herb that is a well-known selenium (Se) and cadmium (Cd) hyperaccumulator which is found in Se-rich environments, and it is narrowly distributed in the wetlands along the Wuling Mountains, China [1, 2, 3, 4, 5]. Other wetland Se accumulators, *Buddleia lindleyana* and *Oenanthe javanica* have also been reported from the same region [6]. In contrast, other Se hyperaccumulators, such as *Opuntia ficus-indica, Stanleya pinnata*, and several *Astragalus* species, occur in arid regions [7, 8, 9, 10]. Several studies have detailed the anatomical structures associated with metal and metalloid hyperaccumulation in plants [1114].

*C. hupingshanensis* plants typically grow to a height of 30–100 cm and thrive in soil containing 0.16-23.74 mg/kg Se [1,5,15]. In a previous study, increased Se concentrations in the soil enhanced the growth and biomass of *C. hupingshanensis* plants under experimental conditions [5,16,17]. A Se soil concentration of 140 mg/kg resulted in the highest biomass accumulation, with higher Se content in the leaves (968 mg/kg) than in the roots (896 mg/kg) or stems (815 mg/kg). At Se concentrations exceeding 140 mg/kg, plant growth was inhibited with reduced biomass, and the leaves and veins were observed to curl and swell, respectively [5,16,17].

Like many other wetland plants, *C. hupingshanensis* is typically subjected to anoxia following flooding [1,18,19,20,21]. Aquatic or amphibious plants have thus evolved various key traits to ensure the survival of the submerged organs, including aerenchyma that store oxygen and apoplastic barriers that impede the escape of air and ions [22, 23, 24, 25, 26, 27, 28, 29, 30, 31].

The aerenchyma and suberized and lignified endodermis and exodermis of the aquatic roots of *Phragmites* and *Oryza* have been extensively studied [18, 20, 22, 23, 26, 27]. The roots of *O. javanica* possess an endodermis and hypodermis with suberin lamellae around the aerenchymal walls [32]. Cortical radial walls with lignified phi (Φ) thickenings (bands of secondary wall thickenings that strengthen the primary wall) have been reported in the roots of certain crops, such as *Brassica napus, B. oleracea*, *Myrica rubra*, *Pyrus malus*, and *Ginkgo biloba* [33, 34, 35, 36, 37, 38]. The amphibious stems of other flood-tolerant plants, including *Cynodon dactylon*, *Hemarthria altissima*, *Paspalum distichum*, *Hydrocotyle sibthorpioides*, *Phalaris arundinacea*, *Typha*, and *Ranunculus trichophyllus* possess a pith cavity and cortical aerenchyma; a cuticle; a suberized and lignified endodermis and exodermis; and a peripheral mechanical ring or periderm [28, 29, 30, 31, 39, 40]. The pith and cortex of the stems of *O. javanica*, *H. altissima*, and *P. distichum* also possess collenchyma [28, 32, 41].

Little information exists on the anatomical and histochemical features of confirmed Se accumulators across various families, including Amaranthaceae, Asteraceae, Brassicaceae, Fabaceae, Rubiaceae, and Orobanchaceae [1, 10]. While various biochemical and physiological analyses have confirmed that *C. hupingshanensis* hyperaccumulates Se and Cd [5, 42], the associated structural and histochemical features of this species are yet to be elucidated. Accordingly, in this study we focused on determining the anatomical features of the roots and shoots of wild-type *C. hupingshanensis* that enable it to hyperaccumulate Se and survive its aquatic lifestyle.

## 2 Materials and Methods

### 2.1 Materials

**Fig. 1:**
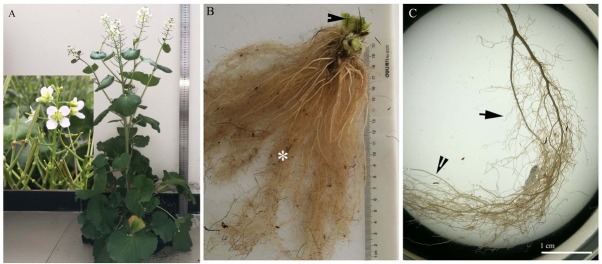
A–C. Morphology of *Cardamine hupingshanensis*. A. Whole plants with flowers and fruits; B. Stems (arrowhead) and adventitious roots (*); C. Fine adventitious root cortex with one or two cell layers, indicated with an arrow (see Fig. 2); taproot cortex with three cell layers (see Fig. 3) and with the cortex sloughed off at the secondary structure, indicated with an arrowhead (see Fig. 4).

The *C. hupingshanensis* samples were collected from the Hupingshan National Natural Reserve in Hunan Province, and from regions of the Yutangba and Liziping in Hubei Province along the Wuling Mountains, China. One-hundred twenty collected samples of *C. hupingshanensis* were preserved in the germplasm resource center of the Hubei Selenium Industry Technology Research Institute, China. Five mature specimens exhibiting normal growth from each collected site, namely Hupingshan, Yutangba, and Liziping, were observed under the microscope. The freshly sampled roots and shoots were fixed in formaldehyde-alcohol-acetic acid (FAA) [43] following collection. After fixing the tissues, freehand sections were cut using a two-sided blade. The sections were made at 5 mm, 15 mm, 30 mm, and 50 mm from the tip to the base with the cortex sloughed off (Fig. 1); through the middle of the stem and the base internode; and through the middle stem petioles and leaves. The sections were about 10 to 25 μm thick.

**Fig. 2:**
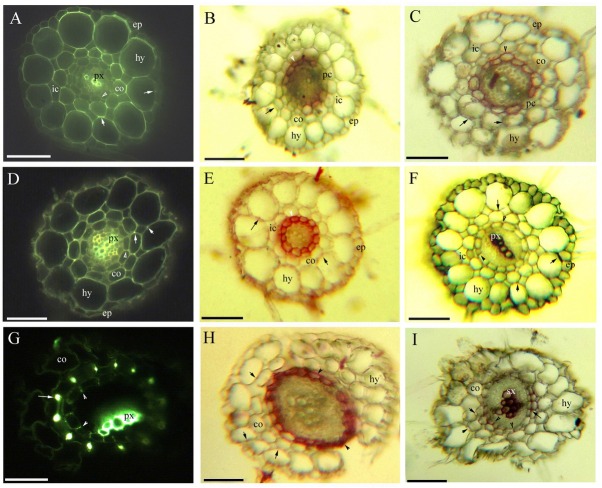
A–I. Photomicrographs of the fine adventitious roots of *C. hupingshanensis* showing some of the secondary growth (50–90 mm long); scale bars = 50 μm; A. Primary xylem, endodermis (arrowhead), cortex, lignified Φ thickenings (arrow), intercellular space, hypodermis, and epidermis. Staining: BAB; B. Endodermis (arrowhead), passage cells, cortex, Φ thickenings (arrow), intercellular space, hypodermis, and epidermis. Staining: SR7B; C. Endodermis (arrowhead), passage cells, cortex, Φ thickenings (arrow), intercellular space, hypodermis, and epidermis. Staining: SR7B; D. Primary xylem, endodermis (arrowhead), cortex, lignified Φ thickenings (arrow), hypodermis, and epidermis. Staining: BAB; E. Endodermis (arrowhead), cortex, Φ thickenings (arrow), intercellular space, hypodermis, and epidermis. Staining: SR7B; F. Primary xylem with metaxylem, endodermis (arrowhead), cortex, lignified Φ thickenings (arrow), intercellular space, hypodermis, and epidermis. Staining: Pg; G. Primary xylem with metaxylem, endodermis (arrowhead), cortex, and lignified Φ thickenings (arrow). Staining: BAB; H. Endodermis (arrow), cortex, Φ thickenings (arrow), and hypodermis. Staining: SR7B; I. Secondary xylem, endodermis (arrowhead), cortex, lignified Φ thickenings (arrow), and hypodermis. Staining: Pg. Abbreviations in the figures are as follows: aerenchyma, ae; Berberine sulfate-aniline blue, BAB; cortex, co; epidermis, ep; cuticle, cu; HCl-phloroglucinol, Pg; pith, pi; hypodermis, hy; intercellular space, ic; parenchyma, pa; passage cells, pc; palisade tissue, pt; primary xylem, px; sclerenchyma ring, sr; Sudan red 7B, SR7B; secondary xylem, sx; toluidine blue O, TBO; vascular bundles, vb;

**Fig. 3:**
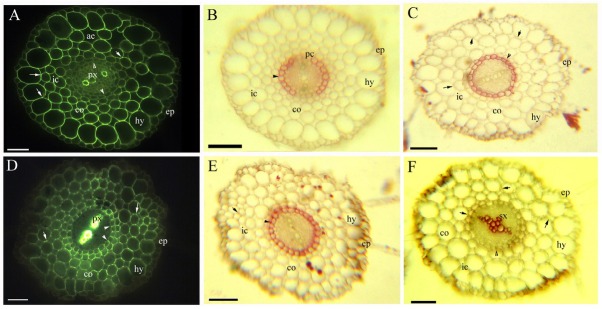
A–F. Photomicrographs of the primary structure of the taproots of *C. hupingshanensis* (120–320 mm long); scale bars = 50 μm; A. Primary xylem, endodermis (arrowhead), cortex, lignified Φ thickenings (arrow), intercellular space, aerenchyma, hypodermis, and epidermis. Staining: BAB; B. Endodermis (arrowhead), passage cells, cortex, intercellular space, hypodermis, and epidermis. Staining: SR7B; C. Endodermis (arrowhead), cortex, Φ thickenings (arrow), intercellular space, hypodermis, and epidermis. Staining: SR7B; D. Primary xylem, endodermis (arrowhead), cortex, lignified Φ thickenings (arrow), hypodermis, and epidermis. Staining: BAB; E. Endodermis (arrowhead), cortex, Φ thickenings (arrow), intercellular space, hypodermis, and epidermis. Staining: SR7B; F. Secondary xylem, endodermis (arrowhead), cortex, intercellular space, lignified Φ thickenings (arrow), intercellular space, hypodermis, and epidermis. Staining: Pg.

**Fig. 4:**
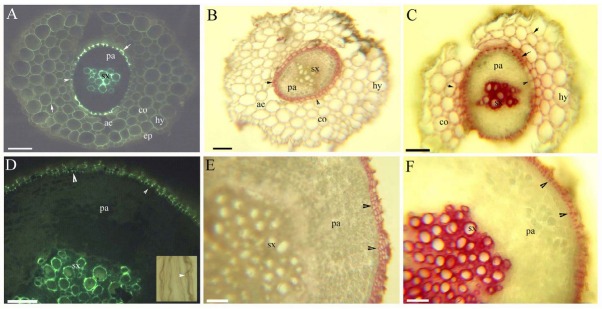
A–F. Photomicrographs of the secondary structure of mature *C. hupingshanensis* taproots (120–320 mm long); scale bars = 50 μm; A. Secondary xylem, parenchyma, endodermis (arrowhead), cortex, lignified Φ thickenings (arrow), aerenchyma, hypodermis, and epidermis. Staining: BAB; B. Secondary xylem, parenchyma, endodermis (arrowhead), cortex, Φ thickenings (arrow), aerenchyma, and hypodermis. Staining: SR7B; C. Secondary xylem, parenchyma, endodermis (arrowhead), cortex, lignified Φ thickenings (arrow), and hypodermis. Staining: Pg; D. Secondary xylem, parenchyma, and periderm Casparian bands (arrowhead). Staining: BAB; inset shows the Casparian bands (arrowhead) on the periderm, sulfuric acid digestion; E. Secondary xylem, parenchyma, and suberized periderm (arrowhead). Staining: SR7B; F. Secondary xylem, parenchyma, and lignified periderm (arrowhead). Staining: Pg.

### 2.2 Methods

All the samples were sectioned under a stereoscope (JNOEC JSZ6, China). For histochemical observation, the sections were stained with Sudan red 7B (SR7B) for the suberin lamellae [31, 44], phloroglucinol–HCl (Pg) for lignin [43], berberine hemisulfate–aniline blue (BAB) for the Casparian bands and thickened cell walls [31, 45,46], and toluidine blue O (TBO) for the other structures including polysaccharides [31,37]. The specimens were examined using bright-field microscopy on a Leica DME microscope and photographed with a digital camera (Nikon E5400, Japan). Specimens stained with BAB were viewed under ultraviolet light on an Olympus IX71 epifluorescence microscope and photographed with a digital camera (RZ200C-21, China).

## 3 Results and Discussion

### 3.1 General morphology

Morphologically, *C. hupingshanensis* is characterized by erect stems (Fig. 1A) and taproots with a mass of fine adventitious roots (Fig. 1B). The fine adventitious roots contain one or two cell cortex layers (Fig. 2). The thick taproots possess three cell cortex layers in the primary structure (Fig. 3), with the cortex sloughed off in the secondary structure (Fig. 1C; Fig. 4).

The fine adventitious roots and young taproots possess a diarch stele with a differentiating proto- and metaxylem; a cortex with an endodermis; and an enlarged outer ring with a distinct hypodermis. The cortex and hypodermal walls have lignified Φ thickenings. Mature taproots possess a typical secondary structure with a periderm.

The stems possess a lignified sclerenchymal ring enclosed within a central cylinder with scattered vascular bundles internal to the cortex, which possesses an endodermis. The pith and cortex walls contain polysaccharide-rich collenchyma. Aerenchyma and intercellular spaces are present in the cortex and pith of the roots and shoots.

### 3.2 Fine adventitious roots

Faint Casparian bands and lightly suberin lamellae and passage cells are present on the endodermis; the cortex and hypodermal walls have slightly lignified Φ thickenings at 5 mm from the root tip (Fig. 2A, B); and the stele has a diarch protoxylem. The endodermis possesses almost complete suberin lamellae and a few passage cells at 15 mm (Fig. 2C). The stele has a metaxylem; the endodermis has faint Casparian bands and lignin, becoming heavily suberized (Fig. 2D, E, F); and the cortex walls have obvious Φ thickenings in the 30 mm section. Narrow intercellular spaces can be observed between the cortex and hypodermis (Fig. 2A–F).

The stele has a few secondary xylems; and the endodermis has obvious and lignified Casparian bands and is heavily suberized at the root base (Fig. 2G, H, I). The inner and radial cortex and hypodermal walls possess both large and small lignified Φ thickenings (Fig. 2G, I); the cortex and hypodermis are partially sloughed off; and the periderm appears suberized (Fig. 2H, I).

### 3.3 Taproots

The stele possesses diarch protoxylem poles; the endodermis has faint Casparian bands and almost complete suberin lamellae with a few passage cells; and the cortex and hypodermal walls have slightly lignified Φ thickenings at 5 mm from the root tip (Fig. 3A, B). The endodermis has almost complete suberin lamellae in the 15 mm section (Fig. 3C).

The stele has a metaxylem and few secondary xylems; the endodermis has Casparian bands and lignin, becoming heavily suberized (Fig. 3D, E, F); and the inner and radial cortex walls have obviously suberized and lignified Φ thickenings at 30 mm. Intercellular spaces and aerenchyma can be observed between the cortex and hypodermis (Fig. 3A–F).

The stele has a secondary xylem; the endodermis has lignified Casparian bands and is heavily suberized at 50 mm (Fig. 4A, B, C). The inner and radial cortex and hypodermal walls possess both large and small lignified Φ thickenings; the cortex and hypodermis have been partially sloughed off; and the periderm is suberized and lignified (Fig. 4A, B, C). The cortex and hypodermis have been sloughed off in the mature taproots; the stele has a secondary xylem and spacious parenchyma; and the periderm has suberized and lignified Casparian bands (Fig. 4D, E, F).

Here we demonstrated that the fine adventitious roots and primary structure of the taproots in the aquatic Se hyperaccumulator *C. hupingshanensis* exhibit similar anatomical and histochemical features. The roots have an endodermis and hypodermis with large cells. The cortex and hypodermal walls have lignified Φ thickenings that are greater near the endodermis. The taproot cortex has more cell layers than the fine adventitious roots, and the mature taproots have a secondary structure containing a periderm, as commonly observed in eudicots.

The young roots of *C. hupingshanensis* are similar in structure to another Se accumulator, *O. javanica* [6, 32]. The hypodermis of *O. javanica* has more cell layers and a cortex with spacious aerenchyma, though the cortex lacks lignified walls and Φ thickenings. Around the roots of *O. javanica* there are aerenchyma, and the walls possess suberin lamellae.

The roots of wetland or aquatic eudicots, such as *R. trichophyllus*, *H. sibthorpioides*, *Artemisia lavandulaefolia*, and *A. selengensis*, possess an endodermis and uniseriate exodermis and a cortex that lacks lignified walls [40, 47,48]. In contrast, the roots of wetland grasses, such as *Phragmites* and *Oryza*, possess an endodermis and multiseriate exodermis [20, 27,28,29,30].

The Φ thickenings of the roots of *C. hupingshanensis* are similar to those of some other brassicaceous species, including *B. oleracea* and *B. napus*, and act as a barrier to ion transport [34, 35]. *Pelargonium hortorum* has larger Φ thickenings at the hypodermis, which is opposite to what is observed in *C. hupingshanensis* [37]. *Myrica rubra*, *P. malus*, and *G. biloba* possess Φ thickenings near the endodermal radial walls but lack lignified walls [33, 36, 38]. Organelle-rich cytoplasm is present in the roots of the nickel (Ni) hyperaccumulator *Senecio coronatus* [1113], and a Cd hyperaccumulating *Arabidopsis thaliana* genotype was found to possess dense root hairs [14]. We believe that lignified Φ thickenings in the roots might trap Se ions and contribute to the Se hyperaccumulation of *C. hupingshanensis*. The dense fine roots and lignified Φ thickenings may allow *C. hupingshanensis* to hyperaccumulate Se in a manner that differs from the Ni hyperaccumulator *S. coronatus* and the Cd hyperaccumulating *A. thaliana* genotype [11,12 14].

The role of air spaces in plant organs is to retain oxygen under hypoxic and anoxic conditions in order to enhance survival [18,19 21,22,23, 26]. The fine roots and primary root structures of the taproots of *C. hupingshanensis* possess fewer intercellular air spaces and aerenchyma than *O. javanica*, *P. distichum*, *P. arundinacea*, *A. lavandulaefolia*, and *A. selengensis* [26, 28,29,30, 32, 40, 47], while *H. sibthorpioides* completely lacks air spaces in the roots [41].

### 3.4 Stems and leaves

**Fig. 5:**
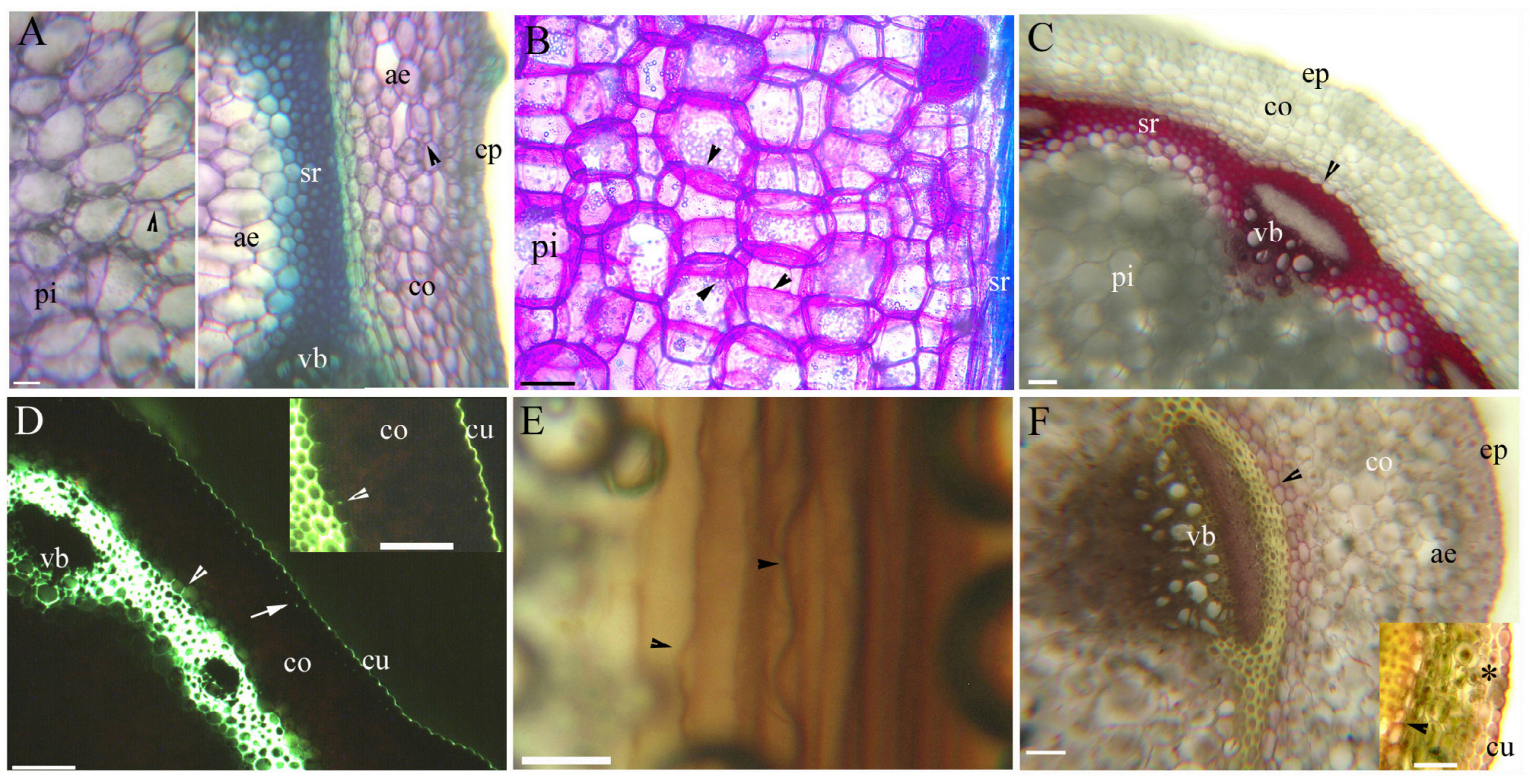
A–F. Photomicrographs of the mature stems of *C. hupingshanensis*. Scale bars = 50 μm. A. Pith, sclerenchymal ring, vascular bundles, cortex, aerenchyma, collenchyma (arrowheads), and epidermis. Staining: TBO; B. Longitudinal sections indicating the pith, sclerenchymal ring, and collenchyma (arrowheads). Staining: TBO; C. Pith, sclerenchymal ring, vascular bundles, endodermis (arrowheads), cortex, and epidermis. Staining: Pg; D. Pith, sclerenchymal ring, vascular bundles, endodermis (arrowheads), cortex, epidermis, cuticle (arrows). Inset is the endodermis (arrowheads), cortex, and cuticle. Staining: BAB; E. Endodermis Casparian bands (arrowheads), sulfuric acid digestion; F. Sclerenchymal ring, vascular bundles, endodermis (arrowheads), cortex, aerenchyma, epidermis. Inset is the endodermis (arrowheads), peripheral mechanical ring (*), and cuticle. Staining: SR7B;

The stems possess a thickened lignified sclerenchymal ring enclosed within a central cylinder with scattered vascular bundles internal to the cortex. The sclerenchymal ring generally has vascular bundles inside it, and a spacious pith is present in the center of the sclerenchymal ring (Fig. 5A, B, C). The cortex has an endodermis with Casparian bands (Fig. 5D, E) and suberin (Fig. 5F) and lignin (Fig. 5C). The outer surface has a cuticle that reaches the inside of the epidermis (Fig. 5D, F). The pith and cortex have aerenchyma, and the walls have unlignified collenchyma that contain polysaccharides (Fig. 5A, B, C, D, F; see also [34]). Beneath the epidermis in the mature stems there is a peripheral mechanical ring (Fig. 5F).

**Fig. 6:**
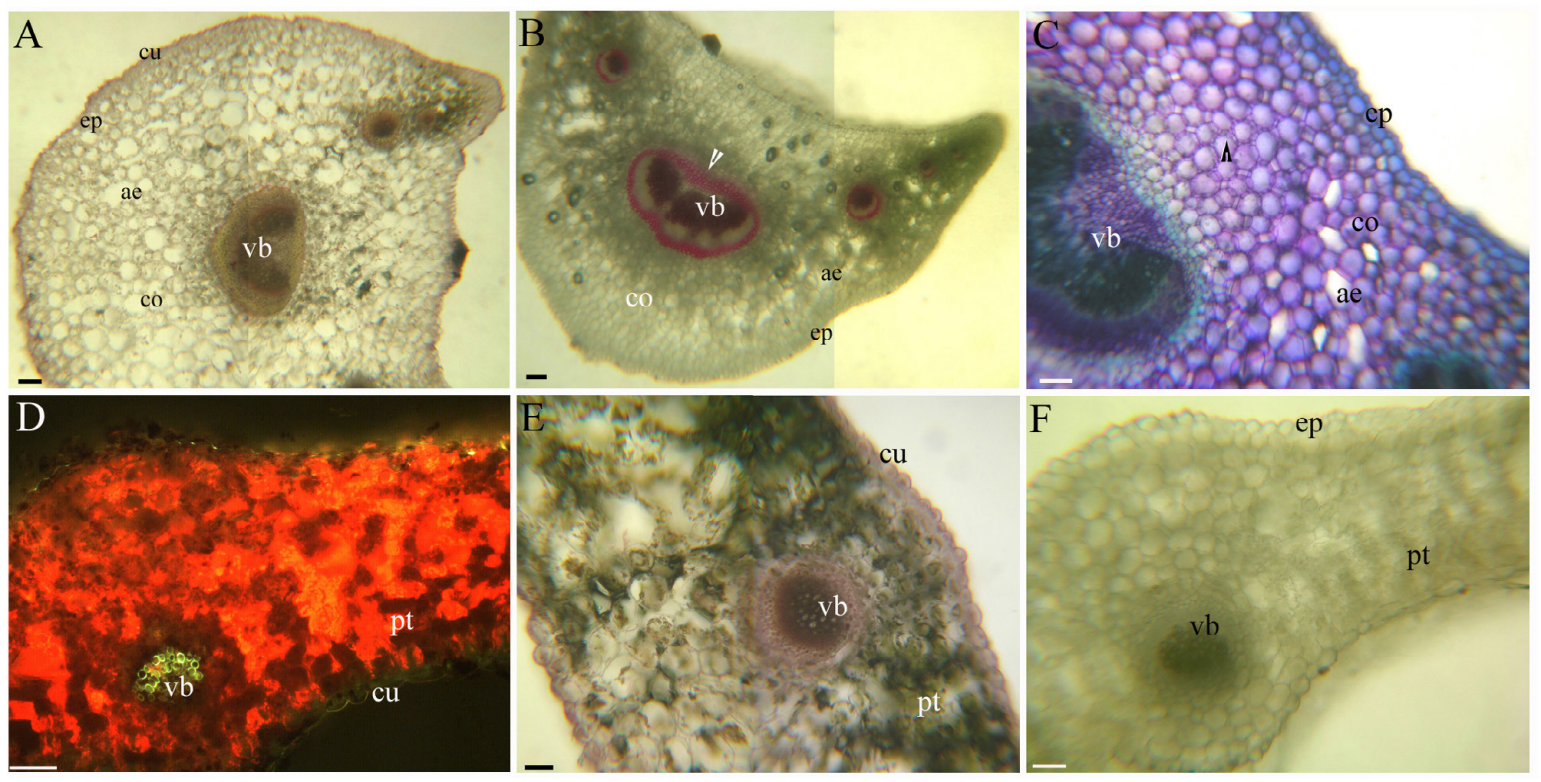
A–F. Photomicrographs of *C. hupingshanensis* leaves. A–C. Transverse sections of the petioles, D–F. Transverse sections of the leaf blades. Scale bars = 100 μm, except C, for which the scale bar = 50 μm. A. Vascular bundles, cortex, aerenchyma, epidermis, and cuticle. Staining: SR7B; B. Vascular bundles, sclerenchymal ring (arrowheads), cortex, aerenchyma, and epidermis. Staining: Pg; C. Vascular bundles, cortex, collenchyma (arrowheads), aerenchyma, and epidermis. Staining: TBO; D. Vascular bundles, palisade tissue, and cuticle. Staining: BAB; E. Vascular bundles, palisade tissue, and cuticle. Staining: SR7B; F. Vascular bundles, palisade tissue, and epidermis. Staining: Pg;

The petioles possess one large and four small vascular bundles with lignified sclerenchymal rings and a spacious cortex with collenchyma and aerenchyma (Fig. 6A, B, C).

A cuticle is present on the surface (Fig. 6A). Cross-sections of the leaf blade reveals vascular bundles, palisade tissue, a cuticle, and vascular bundles that are slightly lignified (Fig. 6D, E, F).

The stems of *C. hupingshanensis* have an endodermis that is similar to *H. sibthorpioides*, *R. trichophyllus*, *P. distichum*, *P. arundinacea*, *A. lavandulaefolia*, and *Typha*, [30, 39,40,41, 47,48]. In contrast, the stems of *P. distichum*, *P. arundinacea*, and *A. lavandulaefolia* possess an exodermis, and the mature stems of *A. lavandulaefolia* and *A. selengensis* have periderm features that are absent in *C. hupingshanensis*. A suberized and lignified peripheral mechanical ring is present in other flood-tolerant species, such as *C. dactylon*, *H. altissima*, *P. distichum*, and *P. arundinacea* [28, 29, 30], as is the lignified sclerenchymal ring observed in *C. hupingshanensis*. However, the collenchyma tissues, which contain polysaccharides, in the pith and cortex walls of *C. hupingshanensis* are larger and thinner than in *H. sibthorpioides*, *H. altissima*, *P. distichum*, and *O. javanica*, whereas the parenchyma are typical of eudicots [28,32,37,48].

The petioles of *H. sibthorpioides* and *R. trichophyllus* have an endodermis, but lack the lignified sclerenchymal ring around the vascular bundles and the cortex with collenchyma that are present in *C. hupingshanensis* [40, 41]. The leaf blade structure of *C. hupingshanensis* is common to eudicots. We speculate that the polysaccharide-rich collenchyma walls of the pith and cortex in the shoots might enhance the tolerance of *C. hupingshanensis* to Se stress.

The cortex and pith of the stems of *C. hupingshanensis* possess a few aerenchyma lacunae, which is similar to that observed in *H. sibthorpioides* following submersion [41]. In contrast, *H. altissima*, *P. distichum*, *A. lavandulaefolia*, and *A. selengensis* have spacious pith cavities that might facilitate survival in heavily submerged conditions [28,29,30, 40, 47]. The stems of *C. hupingshanensis* are similar to those of the typical wetland- and aquatic-adapted plants *H. sibthorpioides* and *R. trichophyllus*, which possess an endodermis [40, 41].

## 4 Conclusion

In summary, *C. hupingshanensis* possesses apoplastic barriers consisting of an endodermis, lignified Φ thickenings, and a cuticle, which is consistent with what has been found in studies on the effects of water stress on oxygen loss and solute transport in plants [18, 19, 22, 23, 24, 25, 32, 41, 45]. The lignified Φ thickenings in the roots and polysaccharide-rich collenchyma in the shoots might have evolved as key structural and histochemical features of *C. hupingshanensis* for Se and Cd hyperaccumulation. These features differ from those of the Ni hyperaccumulator *S. coronatus* and Cd hyperaccumulator *A. thaliana* genotype [2, 5, 11,12, 14, 34,35]. The Se hyperaccumulation of *C. hupingshanensis* might be attributed to the dense fine roots and lignified Φ thickenings, as well as the polysaccharide-rich collenchyma in the shoots. Our results demonstrate that *C. hupingshanensis* possesses anatomical traits that have facilitated its adaptation to the Se-rich, aquatic and subaquatic environments, allowing it to accumulate harmful heavy metal ions and thus remediate contaminated soil [002,3,12,14].

## References

[1] Bai H.F., Chen L.B., Liu K.M., Liu L.H. (2008). A new species of *Cardamine* (Brassicaceae) from Hunan. China. Novon.

[2] Bai H.F., Li X.M. (2012). Cadmium accumulation in hyperaccumulator *Cardamine hupingshanensis*. Jiangsu Journal of Agriculture Science.

[3] Long S.Q., Shao S.X. (2015). Geochemical characteristics of cadmium hyperaccumulator *Cardamine hupingshanensis* in Yutangba. Acta Mineralogica Sinica.

[4] Wang Y.B., Chen F.J., Liang H.W. (2010). A new recorded species of *Cardamine hupingshanensis*. Hubei Agriclture of Science.

[5] Yuan L.X., Zhu Y.Y., Lin Z.Q., Bañuelos G., Li W., Yin X.B. (2013). A novel selenocystine-accumulating plant in selenium-mine drainage area in Enshi. China. Plos One.

[6] Mi X.B., Shao S.X., Tang J., Zhang J. (2014). Identification and characterization of selenium species in Se-enriched plants by HPLC-ICP-MS in Enshi. Pratacultural Science.

[7] Bañuelos G.S., Arroyo I., Pickering I.J., Yang S.I., Freeman J.L. (2015). Selenium biofortification of broccoli and carrots grown in soil amended with Se-enriched hyperaccumulator *Stanleya pinnata*. Food Chemistry.

[8] Bañuelos G.S., Fakra S.C., Walse S.S. (2011). Selenium accumulation, distribution, and speciation in spineless prickly pear cactus: a drought- and salt-tolerant, selenium-enriched nutraceutical fruit crop for biofortified foods. Plant Physiology.

[9] Schiavon M., Pilon-Smits E. A. H (2016). The fascinating facets of plant selenium accumulation–biochemistry, physiology, evolution and ecology. New Phytologist.

[10] White P.J. (2016). Selenium accumulation by plants. Annals of Botany.

[11] Mesjasz-Przybyłowicz J., Barnabas A., Przybyłowicz W. (2007). Comparison of cytology and distribution of nickel in roots of Ni-hyperaccumulating and nonhyperaccumulating genotypes of *Senecio coronatus*. Plant & Soil.

[12] Mesjasz-Przybyłowicz J., Barnabas A., Przybyłowicz W. (2009). Root ultrastructure of *Senecio coronatus* genotypes differing in Ni uptake. Northeastern Naturalist.

[13] Reeves R.D., Baker A.J.M., Jaffré T., Erskine P.D., Echevarria G., van der Ent A. (2018). A global database for plants that hyperaccumulate metal and metalloid trace elements. New Phytologist.

[14] Kohanová J., Martinka M., Vaculík M., White P.J., Hauser M.T, Lux A. (2018). Root hair abundance impacts cadmium accumulation. *Arabidopsis thaliana* shoots. Annals of Botany.

[15] Yang X.L., Li M.L., Yang Y.A., Cao W.S. (2015). Cause analysis of the Tangyanfan geothermal field in Jingshan county,Hubei Province. Resources Environment＆Engineering.

[16] Ming J.J., Yin H.Q., Zhu Y.F., Li W.D., Chen D.Q., Yang Y.D, Yang C.D., Chen F.F., Li Y.J., Ye Z.Y., Wan H.Y., Long L., Wen X.L., Xing J.Q (2017). Effects of selenate stress on the growth physiology of *Cardamine violifolia* O. E. Schulz during seedling stage. Current Biotechnology.

[17] Yan S.K., Zhou S.B., Tao X., Zhang J.Q., Wang L. (2017). Effects of applying selenium on crop yield and quality and Se accumulation in *Cardamine hupingshanensis*. Chinese Journal of Soil Science.

[18] Bailey-Serres J., Voesenek L.A.C.J. (2008). Flooding stress: acclimations and genetic diversity. Annals Review of Plant Biology.

[19] Jackson M.B., Colmer T.D. (2005). Response and adaptation by plants to flooding stress. Annals of Botany.

[20] Kotula L., Ranathunge K., Schreiber L., Steudle E. (2009). Functional and chemical comparison of apoplastic barriers to radial oxygen loss in roots of rice *Oryza sativa* L.) grown in aerated or deoxygenated solution. Journal of Experimental Botany.

[21] Vartapetian B.B., Jackson M.B. (1997). Plant adaptations to anaerobic stress. Annals of Botany.

[22] Armstrong J., Jones R.E., Armstrong W. (2006). Rhizome phyllosphere oxygenation in *Phragmites* and other species in relation to redox potential, convective gas flow, submergence and aeration pathways. New Phytologist.

[23] Colmer T.D., Gibberd M.R., Wiengweera A., Tinh T.K. (1998). The barrier to radial oxygen loss from roots of rice *Oryza sativa* L.) is induced by growth in stagnant solutions. Journal of Experimental Botany.

[24] Enstone D.E., Peterson C.A., Ma F. (2003). Root endodermis and exodermis: structure, function, and responses to the environment. Journal of Plant Growth Regulation.

[25] Meyer C.J., Seago Jr. J.L., Peterson C.A. (2009). Environmental effects on the maturation of the endodermis and multiseriate exodermis of *Iris germanica* roots. Annals of Botany.

[26] Seago Jr. J.L., Marsh L.C., Stevens K.J., Soukup A., Votrubová O., Enstone D.E. (2005). A re-examination of the root cortex in wetland flowering plants with respect to aerenchyma. Annals of Botany.

[27] Soukup A., Armstrong W., Schreiber L., Rochus F (2007). Votrubová O., Apoplastic barriers to radial oxygen loss and solute penetration: a chemical and functional comparison of the exodermis of two wetland species, *Phragmites australis* and *Glyceria maxima*. New Phytologist.

[28] Yang C.D., Zhang X., Zhou C.Y., Seago Jr. J.L. (2011). Root and stem anatomy and histochemistry of four grasses from the Jianghan Floodplain along the Yangtze River. China. Flora.

[29] Yang C.D., Zhang X., Li J.K., Bao M.Z., Ni D.J., Seago Jr. J.L. (2014). Anatomy and histochemistry of roots and shoots in wild rice *Zizania latifolia* Griseb.). Journal of Botany.

[30] Zhang X., Hu L.J., Yang C., Zhou C.Y., Yuan L., Chen Z., Seago Jr. J.L. (2017). Structural features of *Phalaris arundinacea* L. in the Jianghan Floodplain of the Yangtze River, China. Flora.

[31] Zhang X., Hu L.J., Zhou C.Y., Yang C.D. (2017). Comparative study on staining methods and techniques of cell wall histochemistry. Bulletin of Botanical Research.

[32] Zhang X., Hu L.J., Zhou C.Y., Yang C.D. (2016). Studies on anatomy and apoplastic barrier histochemistry characters of *Oenanthe javanica* (Bl.) DC. adapted to wetland Environment. China Vegetables.

[33] Bonacorsi N.K., Seago Jr. J.L. (2016). Root development and structure in seedlings of G*inkgo biloba*. American Journal of Botany.

[34] Fernández-García N., López-Pérez L., Hernandez M., Olmos E. (2009). Role of phi cells and the endodermis under salt stress in *Brassica oleracea*. New Phytologist.

[35] López-Pérez L., Fernández-García N., Olmos E., Carvajal M. (2007). The phi thickening in roots of broccoli plants. An adaptation mechanism to salinity. International Jounal of Plant Science.

[36] Peterson C.A., Emanuel M.E., Weerdenburg C.A. (1981). The permeability of phi thickenings in apple *Pyrus malus* and geranium *Pelargonium hortorum* roots to an apoplastic fluorescent dye tracer. Canadian Journal of Botany.

[37] Peterson R.L., Peterson C.A., Meiville L.H. (2008). Teaching plant anatomy through creative laboratory exercise.

[38] Song Y., Ye L., Nii N. (2011). Effects of soil water availability on development of suberin lamellae in the endodermis and exodermis and on cortical cell wall thickening in red bayberry *Myrica rubra* Sieb. et Zucc.) tree roots. Scientia Horticulturae.

[39] McManus H.A., Seago Jr. J.L., Marsh L.C. (2002). Epifluorescent and histochemical aspects of shoot anatomy of *Typha latifolia* L., *Typha angustifolia* L. and *Typha glauca* Godr. Annals of Botany.

[40] Vecchia F.D., Cuccato F., Rocca N.L., Larcher W., Rascio N. (1999). Endodermis -like sheaths in the submerged freshwater macrophyte *Ranunculus trichophyllus* Chaix. Annals of Botany.

[41] Yang C.D., Li S.F., Yao L., Ai X.R., Cai X.D., Zhang X. (2015). The study on anatomical structure and apoplastic barrier characters of *Hydrocotyle sibthorpioides*. Acta Prataculturae Sinica.

[42] Cui L., Zhao J.T., Chen J.Y., Zhang W., Gao Y.X., Li B., Li Y.F. (2018). Translocation and transformation of selenium in hyperaccumulator plant *Cardamine enshiensis*.

[43] Jensen W.A., Freeman W.H. (1962). Botanical histochemistry – principles and practice.

[44] Brundrett M.C., Kendrick B., Peterson C.A. (1991). Efficient lipid staining in plant material with Sudan red 7B or Fluorol yellow 088 in polyethylene glycol–glycerol. Biotechnology of Histochemistry.

[45] Seago Jr. J.L., Peterson C.A., Enstone D.E., Scholey C.A. (1999). Development of the endodermis and hypodermis of *Typha glauca* Godr. and *T. angustifolia* L. roots. Canadian Journal of Botany.

[46] Brundrett M.C., Enstone D.E., Peterson C.A. (1988). A berberine– aniline blue fluorescent staining procedure for suberin, lignin and callose in plant tissue. Protoplasma.

[47] Zhang X., Yang C.D., Seago Jr. J.L. (2018). Anatomical and histochemical traits of roots and stems of *Artemisia lavandulaefolia* and *A. selengensis* (Asteraceae) in the Jianghan Floodplain. China. Flora.

[48] Yang C.D., Zhang X. (2013). Permeability and supplement structures of stems of *Paspalum distichum*. Bulletin of Botanical Research.

